# Proteotyping Environmental Microorganisms by Phylopeptidomics: Case Study Screening Water from a Radioactive Material Storage Pool

**DOI:** 10.3390/microorganisms8101525

**Published:** 2020-10-04

**Authors:** Karim Hayoun, Olivier Pible, Pauline Petit, François Allain, Virginie Jouffret, Karen Culotta, Corinne Rivasseau, Jean Armengaud, Béatrice Alpha-Bazin

**Affiliations:** 1 Département Médicaments et Technologies pour la Santé (DMTS), CEA, INRAE, SPI, Université Paris Saclay, F-30200 Bagnols-sur-Cèze, France; Karim.Hayoun@cea.fr (K.H.); olivier.pible@cea.fr (O.P.); frallain@gmail.com (F.A.); virginie.jouffret@cea.fr (V.J.); Karen.culotta@cea.fr (K.C.); beatrice.alpha-bazin@cea.fr (B.A.-B.); 2 Laboratoire Innovations technologiques pour la Détection et le Diagnostic (Li2D), Université de Montpellier, F-30207 Bagnols-sur-Cèze, France; 3 CEA, CNRS, INRA, Université Grenoble Alpes, Institut de Biosciences et Biotechnologies de Grenoble, UMR5168, F-38000 Grenoble, France; petitpauline973@gmail.com; 4 CEA-Saclay, DRF/Joliot/SB2SM/BBC, I2BC, 91191 Gif-sur-Yvette, France; corinne.rivasseau@cea.fr

**Keywords:** microorganisms, tandem mass spectrometry, proteotyping, screening, taxonomy

## Abstract

The microbial diversity encompassed by the environmental biosphere is largely unexplored, although it represents an extensive source of new knowledge and potentially of novel enzymatic catalysts for biotechnological applications. To determine the taxonomy of microorganisms, proteotyping by tandem mass spectrometry has proved its efficiency. Its latest extension, phylopeptidomics, adds a biomass quantitation perspective for mixtures of microorganisms. Here, we present an application of phylopeptidomics to rapidly and sensitively screen microorganisms sampled from an industrial environment, i.e., a pool where radioactive material is stored. The power of this methodology is demonstrated through the identification of both prokaryotes and eukaryotes, whether as pure isolates or present as mixtures or consortia. In this study, we established accurate taxonomical identification of environmental prokaryotes belonging to the *Actinobacteria*, *Bacteroidetes*, *Firmicutes*, and *Proteobacteria* phyla, as well as eukaryotes from the *Ascomycota* phylum. The results presented illustrate the potential of tandem mass spectrometry proteotyping, in particular phylopeptidomics, to screen for and rapidly identify microorganisms.

## 1. Introduction

Numerous microorganisms can grow and survive in extreme environments. Life-threatening parameters include, among others, high temperature [1], salinity [2], radiation [3], and desiccation [4]. As adaptability is the essence of the evolution, many strategies have emerged allowing microorganisms to survive in unfavorable conditions. For example, cellular states can be modified through sporulation [5], biofilm formation [6], improved DNA repair mechanisms, and removal of toxic compounds [7]. In this context, the microbial biosphere in extreme habitats represents an interesting reservoir to discover atypical enzymatic catalysts with potential for use in novel biotechnological applications [8,9]. Although many microorganisms are considered uncultivable, the recently-developed culturomics approach, which consists of multiplying the culture conditions to attempt to isolate new microorganisms, has proven successful [10]. However, identification of microorganisms at the different stages of culturomics must be improved to gain faster insights into new branches of the Tree of Life.

The taxonomic identification of novel isolated microorganisms is traditionally achieved by amplifying and sequencing 16S/18S ribosomal RNA (rRNA) genes. However, the resolution of this method is limited, in particular it has difficulty discriminating between closely-related species [11,12], and no universal primers exist covering the whole diversity of microbial life. In addition, the process is time-consuming, making it difficult to include in a screening pipeline. An alternative method, whole-cell matrix-assisted laser desorption/ionization time-of-flight mass spectrometry (MALDI-TOF MS) compares an experimental protein fingerprint against a spectral database established for reference strains. It is quick, and its low operating costs led to its successful integration into clinical diagnostic laboratories [13]. However, the small number of environmental strains available in current MALDI-TOF databases, and the need to have pure isolates for this methodology are considerable drawbacks and have limited its application for environmental samples and screening. A more advanced method, involving tandem mass spectrometry (MS/MS) proteotyping is based on the analysis of peptides generated by trypsin proteolysis of all the proteins extracted from a sample. This methodology can easily discriminate between closely-related species [14] and identify all the components of complex mixtures [15]. In this approach, microorganism identification is generally based on taxon-specific peptides [16]. However, the increasing number of genome sequences has resulted in inflated database sizes, and a consequent decrease in the number of available taxon-specific peptides [17,18]. To overcome this issue, Pible et al. [19] recently described an analytical approach based on the phylopeptidomics signature, a concept through which the number of peptides shared between organisms can be predicted to allow quantification of the relative biomasses of microorganisms in a given sample. Protocols for rapid preparation of bacterial peptides have been proposed to make proteotyping applicable when screening isolates [15,20].

In the present study, we explored the application of phylopeptidomics to screen a broad variety of microorganisms isolated from an industrial infrastructure. Microorganisms were sampled from two radioactive material storage pools and isolated on agar plates. The phylopeptidomics principle is illustrated through several specific cases. Samples identified included a single bacterial isolate, a bacterial mixture, and a eukaryotic isolate.

## 2. Materials and Methods

### 2.1. Microorganism Sampling

Water was sampled from the “Poseidon” and “Osiris” radionuclide storage pools, located at the French Alternative Energies and Atomic Energy Commission (CEA) in Saclay, France. Sampling was performed at the northern wall at 1-m and 4-m depth, the western wall at 1-m and 4-m depth, and the middle of the pool at 1-m and 4-m depth for Poseidon (Pool 1); above the chimney at 5-m depth, inside the chimney at 7-m depth and at the bottom of the pool at 9-m depth for Osiris (Pool 2). In Pool 1, the conductivity, pH and temperature were 140 μS·cm^−1^, pH 7.9 and 23 °C, respectively. The major ions, sodium, potassium, calcium, magnesium, phosphates, and sulphates were below the quantification limit of 5 mg·L^−1^, 0.18 mg·L^−1^, 5 mg·L^−1^, 0.43 mg·L^−1^, 0.2 mg·L^−1^ and 10 mg·L^−1^, respectively. Chlorides and nitrates were at respective mean concentrations of 2.2 mg·L^−1^ and 5.5 mg·L^−1^. In Pool 2, the conductivity, pH and temperature were 0.5 μS·cm^−1^, pH 5.4 and 26 °C, respectively. Microorganisms were harvested through water sampling and concentrated by centrifugation or recovered by filtration through a 0.45-µm cellulose nitrate membrane filter (Sartorius, Göttingen, Germany).

### 2.2. Culture and Isolation

Microorganisms were isolated and cultured under aerobic conditions in the following liquid and agar media: lysogeny broth (LB; BD Bacto), brain heart infusion (BHI; Biomerieux, Marcy l’Etoile, France) and tryptic soy broth (TSB; Biomerieux) diluted 0.1× in water; low-nutrient R2A broth (R2A; [21]) and nutrient broth (NB; 5 g·L^−1^ casein peptones and 3 g·L^−1^ beef extract) diluted 0.5× in water. Colonies were obtained from either: (i) 100 µL of sampled water directly spread onto agar medium, (ii) 100 µL sampled water in 3 mL of liquid media, and the resulting preculture spread onto agar medium, and (iii) cells recovered from filtered water and plated onto agar medium. All cultures were incubated at 22 °C, with agitation at 140 rpm for liquid media, until media became turbid or visible colonies emerged. For proteotyping experiments, colonies were selected based on phenotypic criteria such as color, size and shape, and microorganisms were grown in liquid medium, collected by centrifugation at 8000× *g* for 5 min, and the corresponding pellets were stored at −20 °C until use.

### 2.3. Proteotyping of Isolates by Tandem Mass Spectrometry

Proteins were extracted from the bacterial pellets as previously described by Mappa et al. [22]. Briefly, 1 mg of cell pellet (wet weight) was solubilized in 60 µL lithium dodecyl sulfate (LDS) 1X lysis buffer (Invitrogen, Illkirch, France) supplemented with 5% beta-mercaptoethanol (*v*/*v*). The solution was boiled for 5 min at 99 °C in a thermomixer (Eppendorf, Montesson, France), and sonicated for 5 min in an ultrasonic water bath (ultrasonic cleaner USC 300 T, VWR, Fontenay-sous-Bois, France). Samples were then subjected to bead-beating using a Precellys 24 instrument (Bertin Instruments, Montigny-le-Bretonneux, France) with the following settings: 200 mg silica beads (MP Biomedicals, Illkirch, France) per tube, 3 × 20 s homogenization at 6500 rpm separated by 30-s pauses. Samples were centrifuged at 16,000× *g* for 1 min. Protein extracts were transferred to a new tube and incubated at 99 °C for 5 min. Protein lysates (20 µL) were subjected to a short (5-min) migration on NuPAGE 4–12% Bis-Tris gel (Thermo Fisher). Proteins were proteolyzed in-gel, as described in Hartmann et al. [23]. The polyacrylamide slices containing each proteome were transferred into 96-well plates, reduced and alkylated using 25 mM dithiothreitol in 50 mM NH_4_HCO_3_ at 56 °C for 10 min and 55 mM iodoacetamide in 50 mM NH_4_HCO_3_ for 10 min at room temperature in the dark, respectively. Proteolysis was achieved by addition of 20 µL Trypsin Gold (0.01 µG·µL^−1^, Promega, Charbonnières-les-Bains, France) supplemented with 0.01% ProteaseMAX (Promega) in NH_4_HCO_3_ buffer. ProteaseMAX (50 µL of 0.01% in NH4HCO3 buffer) was added, and samples were incubated at 50 °C for 60 min. The resulting digests were acidified with 5 µL of trifluoroacetic acid (TFA; 0.5% *m*/*v* final concentration). Peptides were analyzed on an LTQ-Orbitrap XL (Thermo Scientific, Waltham, USA) tandem mass spectrometer coupled to an Ultimate 3000 nano LC system (Thermo Scientific) using the parameters previously described (Hayoun et al., 2020). The proteolyzed products were loaded and desalted online on a reversed-phase PepMap 100 C18 μ-precolumn (5 μm, 100 Å, 300 μm i.d. × 5 mm, Thermo Fisher) and resolved on a nanoscale PepMap 100 C18 nanoLC column (3 μm, 100 Å, 75 μmi.d. × 50 cm, Thermo Fisher) at a flow rate of 0.3 μL per min prior to injection into the mass spectrometer. A linear chromatographic gradient of mobile phase A (0.1% HCOOH/100% H_2_O) and phase B (0.1% HCOOH/80% CH_3_CN) was applied. The gradient parameters were as follows: 5% B from 0 to 3 min, and 5–45% B from 3 to 33 min. Full-scan mass spectra were measured from *m*/*z* 350 to 1500 in a data dependent mode using a Top 3 strategy. Briefly, a scan cycle was initiated with a full scan of high mass accuracy in the Orbitrap analyzer operated at 30,000 resolution, which was followed by MS/MS scans in the linear ion trap on the three most abundant precursor ions with a 10-s dynamic exclusion of previously selected ions. Precursor ions were isolated using a 3 *m*/*z* isolation window and activated with 35% normalized collision energy. Minimum signal required was set at 30,000; potential charge states of 2^+^ and 3^+^ were selected. Data were interpreted using Mascot Daemon software version 2.6.1 (Matrix Science, London, UK) with the following parameters: 5 ppm peptide tolerance, 0.05 Da MS/MS fragment tolerance, 2^+^ or 3^+^ peptide charges, one missed cleavage, carbamidomethylation of cysteine as fixed modification, oxidation of methionine as variable modification, and trypsin as proteolytic enzyme. MS/MS spectra were assigned by matching with the NCBInr database (downloaded on 9 November 2015), which contained 76,068,736 protein sequences. No restriction was applied to the taxonomy of the NCBInr database. The phylopeptidomics procedure was applied to identify taxonomies and estimate relative biomass, as previously recommended [19]. Briefly, MS/MS spectra associated with peptide sequences (PSMs) by Mascot were mapped to taxa databases to obtain the raw number of PSMs per taxon (TSMs) at the species, genus, family, order, class, phylum, and superkingdom ranks. For each taxon at each level, the total peptide sequences and total TSMs were counted, as well as specific or unique peptide sequences and corresponding assigned TSMs. The acceptance criteria at the genus level was a minimum of 5 specific peptides, and for a species within a validated genus was at least 50 assigned TSMs and 1 specific peptide. For the validation of an additional species in a given genus, at least 50% of additional assigned TSMs should be recorded compared to the first validated species.

### 2.4. Proteotyping Data

The mass spectrometry proteomics data have been submitted to the ProteomeXchange Consortium via the PRIDE partner repository [24] under dataset identifiers PXD020976 and 10.6019/PXD020976.

## 3. Results

### 3.1. Sampling and Culture Strategies to Obtain Diverse Isolates

Water from two industrial pools located in the CEA Saclay center was sampled.

**Figure 1:**
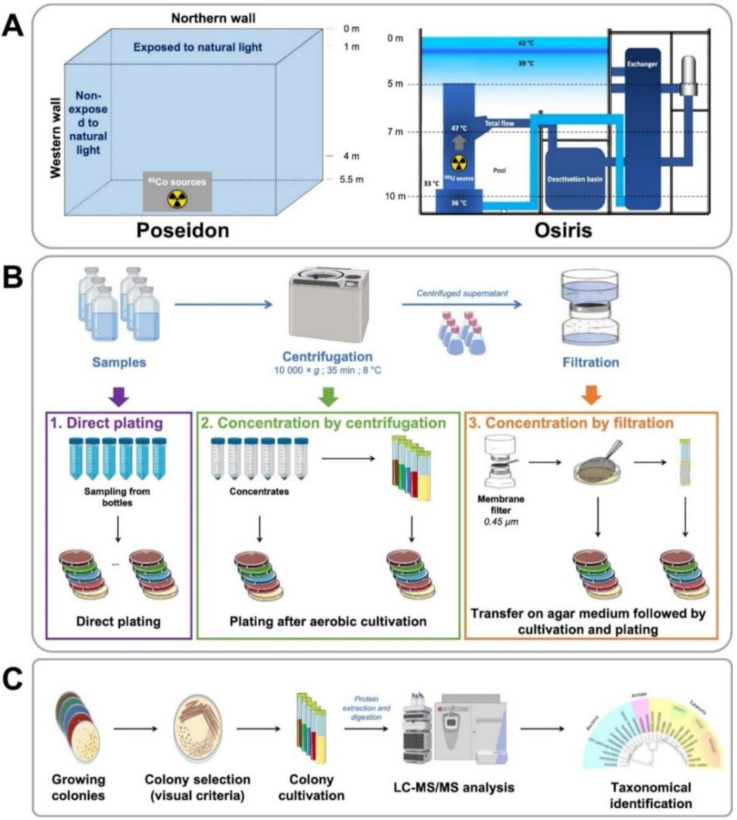
Experimental workflow for microorganism isolation and analysis. (**A**) Schematic representation of the Poseidon and Osiris pools from which the samples were harvested. (**B**) Strategies used to isolate microorganisms, by direct plating, following concentration by centrifugation or filtration. (**C**) Steps involved in tandem mass spectrometry-based identification of microorganisms.

Pool 1 is part of the Poseidon irradiator facility, used to certify nuclear reactor components and sterilize medical materials. The pool is located in a containment chamber and is used to store a single Cobalt 60 source at the bottom of the pool, as illustrated in Figure 1A (left panel). At the time of sample collection, the radiological control indicated a level of alpha and beta radiation below the limits of detection. Water samples were collected at several locations in the pool, at various depths and levels of exposure to natural light.

Pool 2 is within the Osiris nuclear reactor, which was used until recently to produce radionuclides for medicine from radioactive Uranium 235 sources (Figure 1A, right panel). Water was sampled at three separate points located at different distances from the radioactive source location. The irradiation rates recorded at the time of collection, i.e., one year after the reactor was shut down, ranged between <1 and 25 µGy·h^−1^.

The strategy for microorganism culture is presented in Figure 1B. To obtain diverse microbial isolates, microorganisms were either isolated directly by plating sampled water on R2A 0.5× and TSB 0.1× agar plates or concentrated by centrifugation or filtration prior to culture. Concentrated samples were either directly plated on LB 0.1×, R2A 0.5×, BHI 0.1×, TSB 0.1× and NB 0.5× agar media, or first enriched by liquid culture from filters in R2A 0.5× medium. Microorganisms were chosen depending on visible colony criteria (color, shape, size) (Figure 1C). The proteomes of the harvested cells were analyzed by tandem mass spectrometry, and data were exploited to establish their taxonomical identity.

### 3.2. How Phylopeptidomics Can Be Used to Verify Whether a Sample is Mono-Organism or Contains a Mixture of Microorganisms

**Figure 2:**
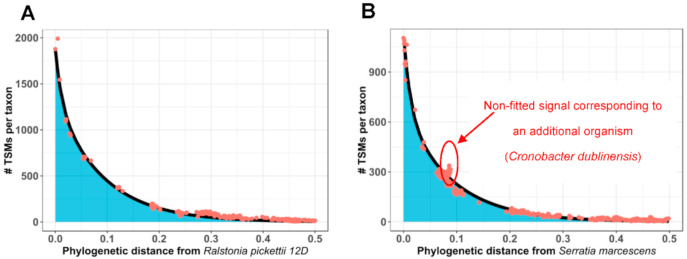
Mathematical fitting of two different bacterial samples with a unique phylopeptidomics signature, illustrating the correct fit of a mono-organism isolate (**A**), and the degraded fit obtained when a sample containing a mixture of two bacterial strains is fitted with a single signature (**B**). The orange dots indicate the referenced taxa present in the database while the black curve represents the theoretical exponential distribution of taxon-spectrum matches (TSMs).

The phylopeptidomics signature, which was reported as a new means to quantify the relative biomasses of organisms in Pible et al. [19], is also a powerful means to inspect, both visually and computationally, the nature of the sample from which the MS/MS spectra were acquired. The signature concept is based on modeling of the signal for a pure organism using a mathematical bi-exponential formula, known as the phylopeptidomics signature. This signature can perfectly fit the proteomics signal when plotted against an *X*-axis representing inter-taxa distances based on conserved proteins. In this representation, the proteomics signal is expressed as taxon-spectrum matches (TSMs) for each organism present in the database. For a pure microorganism, the signal will be observed to decrease as the phylogenetic distance between organisms from the database and the correct taxonomical group increases. Mixtures of several organisms can be modeled using a linear combination of signatures, to yield relative biomass quantification. Thus, the purity of an isolate can readily be assessed by checking if a unique signature fits the whole signal. An example is shown in Figure 2, where a mono-signature is fitted both to a single-organism sample (panel A), and a mixture of two organisms, isolated from another microbiota (panel B). Visual examination and a sum of errors check agree that fitting is inadequate for panel B, because (i) the missing signature offsets the mono-organism fit, and (ii) a collective upward signal corresponds to the non-fitted organism.

### 3.3. Examples of Species-Level Identification and Biomass Quantification for Mixtures of Microorganisms

**Figure 3:**
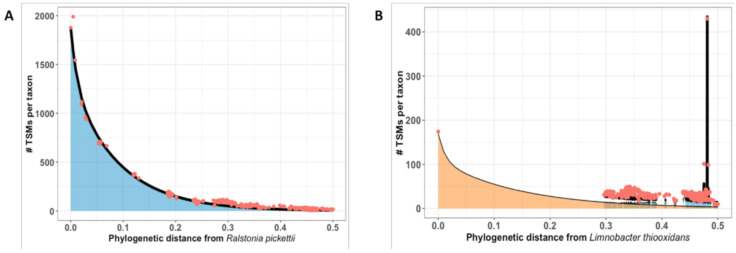
Phylopeptidomics signatures for the *R. pickettii* 12D pure isolate (**A**) and the *N. ramosa* DSM 11499 and *L. thiooxydans* mixture represented by orange and blue area, respectively (**B**). Plots show the number of TSMs assigned (*Y*-axis) for the microorganisms present in the database, ranked based on their phylogenetic distance from the most abundant microorganism (*X*-axis). The thin black curve represents the theoretical exponential distribution of TSMs. The thick black line corresponds to the sum of phylopeptidomics signatures. The red dots represent taxa referenced in the database.

Among the samples characterized by phylopeptidomics, sample A (Osiris pool, water sampled at 9-m depth) and sample B (Poseidon pool, water sampled at 1-m depth at the western wall) from Figure 3 were shown to correspond to a unique bacterium and a mixture of two bacterial species, respectively. For these two samples, the numbers of MS/MS spectra recorded were 3988 and 3293, respectively (Table S1). Matching sequences against the NCBInr database allowed the interpretation of 51% and 23% of these spectra, respectively. The lower ratio of spectra assigned for sample B could be due to either (i) its higher complexity as the mixture of two microorganisms may lead to mixed MS/MS spectra that are difficult to assign, or/and (ii) the genomic divergence between the microorganisms present in the samples compared to those genome sequenced and present in the general database. Nevertheless, peptide sequences allowed accurate taxonomical assignments based on TSMs. The numbers of TSMs and taxon-specific peptides are listed at the various taxonomical levels, i.e., Superkingdom, Phylum, Class, Order, Family, Genus, and Species (Table S1).

The MS/MS-derived information identified sample A as *Ralstonia pickettii*, based on 130 species-specific peptides. Figure 3A shows the phylopeptidomics signature at the species level for this single bacterium. The graph indicates the number of TSMs for all the bacteria present in the database ranked along the *X*-axis based on their phylogenetic distance from *R. pickettii*. This representation shows a strong correlation between the experimental data and the theoretical phylopeptidomics signature fit calculated for a pure *R. pickettii* sample. This profile matching confirms that the sample corresponds to the *R. pickettii* species, and that the sample contains a single organism.

Sample B was found to contain two distinct species, based on identification of their taxon-specific peptides. The two species were *Nevskia ramosa* and *Limnobacter thiooxidans*, identified by 143 and 73 species-specific peptides, respectively. The experimental TSMs from the *N. ramosa* and *L. thiooxidans* mixture are distributed among the microorganisms present in the database as the sum of their respective phylopeptidomics signatures. Figure 3B shows the perfect match obtained between the phylopeptidomics signature for experimental *N. ramosa* data and the theoretical signature. The additional signal can be explained by the presence of the phylopeptidomics signature for L. thiooxidans. Moreover, after deconvolution of the two signatures, phylopeptidomics provides information on the relative abundances of the different strains in the sample. In this case, *N. ramosa* and *L. thiooxidans* were estimated to be present at 71% and 29%, respectively.

With Pible et al. (2020), for formalism, each dot on Figure 3B corresponds to a taxon *i,* with « # TSMs per taxon » quantified as the sum of two signatures:

(1) $$\begin{array}{cc} y_{i,REF1}+\;y_{i,REF2} & =N_{REF1}\;\times \;\left(A_{REF1}\times e^{-\;\frac{x_{i,REF1}}{a_{REF1}}}+\left(1-A_{REF1}\right)\times e^{-\;\frac{x_{i,REF1}}{b_{REF1}}}\right)\\ & +\;N_{REF2}\times \;\left(A_{REF2}\times e^{-\;\frac{x_{i,REF2}}{a_{REF2}}}+\left(1-A_{REF2}\right)\times e^{-\;\frac{x_{i,REF2}}{b_{REF2}}}\right) \end{array}$$

with the following parameters: *REF*1 = *Limnobacter thiooxidans*, *A_REF_*_1_ = 0.22637, *a_REF_*_1_ = 0.012, *b_REF_*_1_ = 0.14035, *N_REF_*_1_ = 180.3, *X_i_,_REF_*_1_ = phylogenetic distance between taxon *i* and *REF*1; and *REF*2 = *Nevskia ramosa*, *A_REF_*_2_ = 0.15336, *a_REF_*_2_ = 0.012, *b_REF_*_2_ = 0.14928, *N_REF_*_2_ = 434.0, *X_i_,_REF_*_2_ = phylogenetic distance between taxon *i* and *REF*2.

The numbers of species-specific peptides are not representative of the quantities of the two bacteria, as they depend mainly on the density of genome sequences available in the database searched for the two corresponding branches of the Tree of Life [19]. In other samples, we also identified *L. thiooxydans* (Sample J) and *N. ramosa* (Sample N) as pure isolates. These two examples demonstrate how phylopeptidomics can determine whether a sample is a mixture of microorganisms, and if so, how it can decipher their relative biomasses.

### 3.4. Phylopeptidomics also Successfully Identifies Fungi

**Figure 4:**
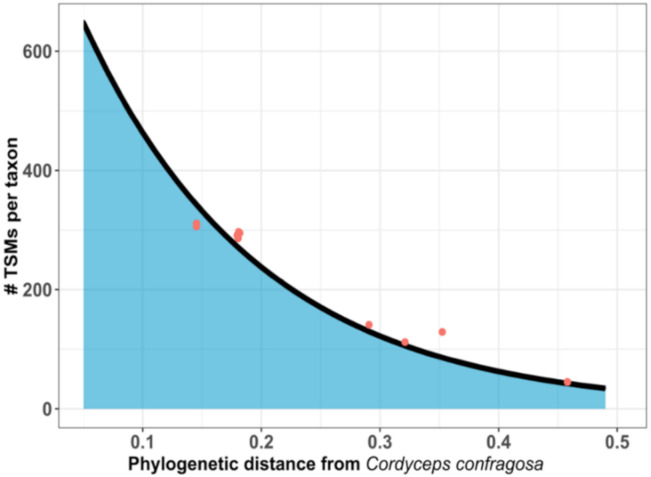
Phylopeptidomics signature for *C. confragosa* RCEF 1005 MS/MS dataset. The *Y*-axis represents the number of TSMs assigned for each microorganism present in the database, whereas the *X*-axis shows the phylogenetic distance between these microorganisms and the organism identified. The black curve represents a theoretical exponential distribution of TSMs relative to the phylogenetic distance separating organisms, with the red dots corresponding to the taxa referenced in the database.

A total of 2740 MS/MS spectra were recorded for sample C. Querying against the NCBInr database resulted in assignment of 33% of these spectra (PSMs). The taxon-specific peptides indicate the presence of one *Ascomycota* belonging to the *Cordycipitaceae* family: *Cordyceps confragosa*. The database used for this analysis comprises the theoretically genome-derived proteomes from 200 members of this family distributed across 11 distinct genera: *Akanthomyces, Ascopolyporus, Beauveria, Cordyceps, Gibellula, Hyperdermium, Isaria, Lecanicillium, Microhilum, Simplicillium*, and *Torrubiella*. Due to the pleomorphic nature of fungi and some taxonomical confusion, this family was recently reclassified in terms of molecular phylogeny [25]. Here, 44 species-specific peptides were assigned to *Cordyceps confragosa*, which is known to be a pathogen of scale insects, and the abundance signal was high (685 TSMs). The phylopeptidomics signature for this dataset is shown in Figure 4 and indicates that the sample contains a pure species. Fitting of the phylogenetic signature produced a unique exponential curve, representing as expected the decrease in TSMs as the distance between the microorganisms present in the database and *C. confragosa*—for which the highest number of TSMs was assigned—increased. *C. confragosa* is considered to be part of the *Lecanicillium* genus (*Akanthomyces*) as discussed recently in Kepler et al. [25], and differs significantly from *Cordyceps militaris*, belonging to the *Cordyceps* genus, and *Cordyceps brongniartii*, which is associated with the *Beauveria* clade. For these microorganisms, 558 and 556 TSMs were counted, respectively. These TSMs can be explained by the large proportion of sequences shared between these fungal strains, but other taxa in this genus were not validated due to the low number of taxon-specific peptides for *C. militaris* (11) and *C. brongniartii* (2) compared to *C. confragosa* (44), and consequently their low specifically assigned TSMs (13 and 2, compared to 685, respectively). This example illustrates how tandem mass spectrometry is powerful in discriminating the correct branches of the Tree of Life while some families are wrongly characterized and deserve phylogenetic reclassification.

### 3.5. Analysis of the Whole Cohort of Microorganisms Identified

After tandem mass spectrometry proteotyping and dereplication which consists of removing samples with the same species assignation, a set of 29 samples were further investigated. Table 1 shows the results for these samples in terms of species identified, number of TSMs, and number of taxon-specific peptides for the Species and Genus taxonomical ranks. Table S1 presents the specific peptides and TSMs at all the other taxonomical ranks, as well as the water sample origin and characteristics. A mean of 3125 (±35%) MS/MS spectra were recorded for these samples, with on average 738 (±47%) MS/MS spectra from each sample assigned to peptide sequences. In some cases, the recorded signal was low, as for the example of *Methylobacterium extorquens* with only 427 MS/MS spectra identified. The identification was nevertheless crystal-clear as the number of species-specific peptides was significant. All the isolates (21) were accurately identified at the species level with a significant number of TSMs and taxon-specific peptides assigned. For the remaining eight samples, we report the identification of mixtures of microorganisms. These results led to the identification of a total of 29 individual microorganisms at the species level. Among these, the prokaryotic microorganisms identified included 4 *Actinobacteria*, 2 *Bacteroidetes*, 4 *Firmicutes*, and 19 *Proteobacteria*. In addition, two fungi belonging to the *Ascomycota* phylum were detected: *Cladosporium herbarum* and *Cordyceps confragosa*. Four of the samples reported in Table 1 originated from the Osiris pool (samples A, R, S, and AB). This relatively low number was due to the small number of microorganisms present in this environment compared to the Poseidon pool.

## 4. Discussion

The present study illustrates how phylopeptidomics can be applied to rapidly and reliably taxonomically identify microorganisms. The results presented in this article show that MS/MS proteotyping can be successfully applied to environmental bacterial and fungal isolates. The approach can determine whether a sample is mono-organism or if it is a mixture of microorganisms requiring further isolation efforts.

The MS/MS methodology applied here was recently used to identify bacteria isolated from human dental swabs, and results compared favorably with whole-cell MALDI-TOF mass spectrometry typing [15]. For this specific dataset, only 2 out of 24 bacterial isolates could be identified at the species taxonomical rank by the MALDI-TOF-based method, whereas all were identified by MS/MS proteotyping. With the new study presented here, the next-generation methodology was shown to efficiently identify isolates belonging to very different branches of the Tree of Life, for which reference MALDI-TOF spectra are not yet available. The MALDI Biotyper CA System which has been recently granted USA Food and Drug Administration clearance can identify 333 species or species groups, covering 424 clinically relevant bacteria and yeast species. While this system is adapted for the great majority of bacteria analyzed in the clinical microbiology laboratories, it is not worth it for environmental screening. As indicated by Pible et al. [19], the phylopeptidomics signature takes the whole Tree of Life into account, modeling the distribution of shared peptides across all the known organisms distributed according to their phylogenetic distance from the microorganism identified. It can thus be applied to any microorganism, covering bacteria, archaea, and eukaryotic phyla. To our knowledge, no other identification methodology has such a wide applicability in terms of types of microorganisms identified. For example, 16S/18S RNA amplicon sequencing must be performed with specific sets of primers depending on whether the microorganisms are bacteria or eukaryotes. Whole-cell MALDI-TOF mass spectrometry requires specific sample preparation, especially for fungi, combined with specific databases. Its applicability for de novo high-throughput taxonomical identification is therefore limited. The protocol applied here to extract proteins prior to tandem mass spectrometry is generic—applicable to any microorganism present in the sample—and, as previously shown, works equally well on vegetative cells and spores [22]. The results presented here show MS/MS proteotyping to be potentially universal, and applicable even with mixtures of microorganisms. Based on the large number of taxon-specific peptides obtained for each isolate, the confidence of identification is high. As recently shown, this MS/MS proteotyping approach is superior to whole-cell MALDI-TOF mass spectrometry for environmental isolates [15]. Thus, results need not be confirmed with either 16S/18S RNA amplicon sequencing or whole-cell MALDI-TOF mass spectrometry.

To identify the correct species, a closely related genome must be present in the database, along with up-to-date taxonomy information. Extensive efforts have been made to explore what is known as microbial dark matter, by investigating novel microbial lineages based on molecular information [26,27]. Here, the general database used comprises genomic information for 15,335 species, representing information for 76,068,736 protein sequences. It thus represents a broad panorama of microbial life on Earth. However, this database is far from comprehensive, and we expect it to be further improved in the coming years as new genome sequences become available, resulting in a better coverage of the Tree of Life.

For validating the identification at a given taxonomical level, we have considered a conservative approach based on the number of specific peptides and abundance assessed by TSMs. However, confidence of identification would be better estimated with individual spectrum matches at the peptide level combined into a single statistical measure at each of the taxonomical levels. For this, specific statistical developments should be proposed and assessed with experimental data acquired on reference species from different branches of the Tree of Life used as standards.

For a large number of environmental samples, specific genera are only known through a single reference species for which the genome has been sequenced. In this case, all the isolates from these branches of the Tree of Life will be assimilated to the corresponding reference species. None of the microorganisms identified in this study (Table 1) belong to this type of theoretical case, as genome sequences were available for several representatives of each genus identified and were included in the general NCBInr database used for query matching. For example, the genus *Rheinheimera*, generally found in water streams, coastal sediments, and soils, which is not very well characterized per se [28], was represented by 23 genomes corresponding to 11 distinct species. These figures indicate that the microorganisms isolated from the water samples are mostly assigned to well-known branches of the Tree of Life and correspond to easily cultivable environmental microorganisms.

Another interesting proteotyping case may come from a taxonomical group for which no reference species has yet been sequenced, but for which genomes are available only for non-taxonomically assigned isolates. In the present study, this was the case for *L. thiooxydans*, as six *Limnobacter* sp. strains related to this species have been genome sequenced, but none of which has been designated as the reference strain of any new species. In this case, matches are readily assigned to the reference species name. However, further investigations to more appropriately classify these specific taxa will be needed to obtain more accurate identification. Efforts of this type will be essential to improve identification methods, whatever the methodology used, and to refine meta-omics analysis in general as previously discussed [29]. In the case of MS/MS proteotyping, any increase in the number of genomes that can be included in the general database will improve the overall quality of the taxonomical identifications. It is thus worth deploying efforts to increase the number of genomes of less well-represented branches of the Tree of Life, for which little sequencing information is currently available, to improve the taxonomical classification of unknown microorganisms [30]. To extend our knowledge, better microbial isolation procedures, such as the combination of culturomics and MS/MS proteotyping to rapidly identify the least-characterized microorganisms, should be proposed.

Whereas most of the samples were abundant in terms of biological material, a very small amount of protein was extracted from the sample identified as *Methylobacterium extorquens*. This was due to the low number of cells obtained, possibly due to non-optimal medium or culture conditions. Consequently, only 427 MS/MS spectra were recorded for this sample. However, this low signal did not hamper correct identification, as 26 genus-specific peptides and 3 species-specific peptides were confidently identified. This example shows that MS/MS proteotyping is sensitive and could be incorporated in culturomics strategies based on microfluidics dealing with small culture volumes. As protein extracts and peptides can be prepared in 96-well plates [15], and subsequent mass spectrometry and interpretation steps are compatible with automation, the methodology is well worth implementing in culturomics laboratories. Tandem mass spectrometry-based proteotyping is currently more costly than MALDI-TOF and requires expertise and infrastructure that are available only in specific platforms. These drawbacks should be carefully evaluated with the power of the approach in mind. Miniaturizing the required instruments, excogitating the potential of the approach amongst microbiologists, teaching the corresponding know-how, and further benchmarking are necessary actions to spread this methodology.

Classical agar plating methods have been shown to be relatively limited for the culture of most microorganisms from environmental samples [31]. Here, cultivation in low-nutrient media was attempted to favor some slow-growing microorganisms, and during this proof of concept study, we isolated several microorganisms. The set of microorganisms identified, 4 *Actinobacteria*, 2 *Bacteroidetes*, 4 *Firmicutes*, 26 *Proteobacteria*, and 2 *Ascomycota*, shows some diversity, but there is nevertheless a strong predominance of *Proteobacteria*. The relatively rich media used in the study probably explains this significant dissymmetry, but further large-scale culturomics studies should allow isolation of microorganisms belonging to poorly characterized branches of the Tree of Life. Other studies have investigated the cultivable microbial diversity in nuclear fuel storage facilities by growing isolates on classical media and identified species by 16S RNA amplicon sequencing. They identified similar genera to those detected here. For example, Chicote et al. [32] reported the presence of six bacterial strains—including *Ralstonia* and *Bacillus* genera—and a eukaryotic fungus, *Aspergillus fumigatus*. Another study on biofilm development in spent-nuclear-fuel pools revealed the presence of *alpha, beta,* and *gamma-Proteobacteria, Actinobacteria*, and *Firmicutes* [33]. Meta-analyses of our own data also revealed the predominance of an alpha-*Proteobacteria* (*Methylobacterium*) in the Osiris pool (Petit, Armengaud, Rivasseau, unpublished data). These microorganisms could have interesting biotechnological applications, as suggested by several other studies. Thus, for example, the green microalgae *Coccomyxa actinabiotis* was isolated from a nuclear infrastructure [34], and the uranium-tolerant *Microbacterium oleivorans* A9 was isolated from Chernobyl-contaminated soil [35].

In conclusion, the results obtained here highlight the potential of tandem mass spectrometry proteotyping and phylopeptidomics to taxonomically characterize environmental isolates, whether prokaryotic or eukaryotic, mono-cultures, or more complex mixtures. This approach is highly recommended to improve the output from culturomics strategies.

## Figures and Tables

**Table 1:** Proteotyping results for the 29 samples * extensively analyzed.

Sample	MS/MS Spectra	Total PSMs	Organism(s)	Tax ID	Species-Assigned TSMs	Species-Specific Peptides	Genus-Specific Peptides
**A**	3988	2020	*Ralstonia pickettii*	329	1990	130	338
**B**	3293	750	*Nevskia ramosa*	64002	430	143	158
			*Limnobacter thiooxidans*	131080	150	73	73
**C**	2740	907	*Cordyceps confragosa*	1105325	685	44	74
**D**	2951	576	*Bacillus cereus*	1396	514	2	33
**E**	3001	747	*Bacillus indicus*	246786	580	2	174
**F**	3369	1210	*Bacillus thuringiensis*	1428	1195	43	221
**G**	3223	738	*Bosea vaviloviae*	1526658	465	25	246
**H**	4135	638	*Brevundimonas bacteroides*	74311	436	29	275
**I**	1955	364	*Hymenobacter swuensis*	1446467	296	48	189
**J**	4264	943	*Limnobacter thiooxidans*	131080	861	418	418
**K**	3765	1350	*Massilia timonae*	47229	1283	516	656
**L**	427	106	*Methylobacterium extorquens*	408	67	3	26
**M**	3356	846	*Microbacterium oxydans*	82380	771	47	466
**N**	3407	460	*Nevskia ramosa*	64002	355	123	140
**O**	3007	868	*Pantoea vagans*	470934	758	13	102
**P**	3871	621	*Porphyrobacter cryptus*	196588	410	38	162
**Q**	3479	1259	*Propionibacterium acnes*	1747	1228	15	585
**R**	4142	1111	*Sphingomonas echinoides*	59803	1009	231	607
**S**	4139	835	*Sphingomonas elodea*	179878	668	97	362
**T**	3619	1214	*Sphingomonas parapaucimobilis*	28213	934	180	713
**U**	1352	262	*Xanthobacter autotrophicus*	280	144	13	29
**V**	1509	153	*Cladosporium herbarum*	29918	12	7	7
**W**	2209	316	*Limnobacter thiooxidans*	131080	94	55	55
			*Bacillus*	1386	133	/	40
			*Hymenobacter norwichensis*	223903	40	20	31
**X**	2054	496	*Rhodococcus erythropolis*	1833	338	4	179
			*Massilia timonae*	47229	97	49	61
**Y**	3451	661	*Sphingomonas parapaucimobilis*	28213	182	10	129
			*Caulobacter vibrioides*	155892	160	33	72
			*Limnobacter thiooxidans*	391597	113	54	54
**Z**	3972	454	*Variovorax paradoxus*	34073	194	2	7
			*Acidovorax*	12916	95	/	5
			*Polaromonas*	52972	33	/	6
**AA**	3760	516	*Rheinheimera texasensis*	306205	324	35	87
			*Limnobacter thiooxidans*	131080	37	23	23
**AB**	1840	356	*Cellulomonas gilvus*	11	86	1	23
			*Sphingomonas parapaucimobilis*	28213	137	11	77
**AC**	4349	623	*Xanthobacter autotrophicus*	280	357	23	70
			*Azorhizobium caulinodans*	7	52	3	9

* Samples A, R, S and AB were obtained by direct plating water collected from pool 2; samples B, N and P were obtained by direct plating water collected from pool 1; samples C, D, E, F, H, I, J, K, L, M, O, T, V, W, Y, Y, and Z were obtained from water collected from pool 1 and concentrated by filtration before plating; samples G, Q, U, AA and AC were obtained from water collected in pool 1 enriched by centrifugation before plating.

## Supplementary Materials

The following are available online at https://www.mdpi.com/2076-2607/8/10/1525/s1, Table S1. Proteotyping results for the 29 samples analyzed extensively.

## References

[1] Togawa Y., Shiotani S., Kato Y., Ezaki K., Nunoshiba T., Hiratsu K. (2019). Development of a supF-based mutation-detection system in the extreme thermophile Thermus thermophilus HB27. Mol. Genet. Genom..

[2] Rubiano-Labrador C., Bland C., Miotello G., Guerin P., Pible O., Baena S., Armengaud J. (2014). Proteogenomic insights into salt tolerance by a halotolerant alpha-proteobacterium isolated from an Andean saline spring. J. Proteom..

[3] Zivanovic Y., Armengaud J., Lagorce A., Leplat C., Guerin P., Dutertre M., Anthouard V., Forterre P., Wincker P., Confalonieri F. (2009). Genome analysis and genome-wide proteomics of Thermococcus gammatolerans, the most radioresistant organism known amongst the Archaea. Genome Biol..

[4] De Groot A., Dulermo R., Ortet P., Blanchard L., Guerin P., Fernandez B., Vacherie B., Dossat C., Jolivet E., Siguier P. (2009). Alliance of proteomics and genomics to unravel the specificities of Sahara bacterium Deinococcus deserti. PLoS Genet..

[5] McKenney P.T., Driks A., Eichenberger P. (2013). The Bacillus subtilis endospore: Assembly and functions of the multilayered coat. Nat. Rev. Microbiol..

[6] Tolker-Nielsen T. (2015). Biofilm Development. Microbiol. Spectr..

[7] Hermon L., Denonfoux J., Hellal J., Joulian C., Ferreira S., Vuilleumier S., Imfeld G. (2018). Dichloromethane biodegradation in multi-contaminated groundwater: Insights from biomolecular and compound-specific isotope analyses. Water Res..

[8] Armengaud J. (2016). Next-generation proteomics faces new challenges in environmental biotechnology. Curr. Opin. Biotechnol..

[9] Kruger A., Schafers C., Schroder C., Antranikian G. (2018). Towards a sustainable biobased industry-Highlighting the impact of extremophiles. New Biotechnol..

[10] Lagier J.C., Khelaifia S., Alou M.T., Ndongo S., Dione N., Hugon P., Caputo A., Cadoret F., Traore S.I., Seck E.H. (2016). Culture of previously uncultured members of the human gut microbiota by culturomics. Nat. Microbiol..

[11] Brandt J., Albertsen M. (2018). Investigation of Detection Limits and the Influence of DNA Extraction and Primer Choice on the Observed Microbial Communities in Drinking Water Samples Using 16S rRNA Gene Amplicon Sequencing. Front. Microbiol..

[12] Rutanga J.P., Van Puyvelde S., Heroes A.S., Muvunyi C.M., Jacobs J., Deborggraeve S. (2018). 16S metagenomics for diagnosis of bloodstream infections: Opportunities and pitfalls. Expert Rev. Mol. Diagn..

[13] Grenga L., Pible O., Armengaud J. (2019). Pathogen proteotyping: A rapidly developing application of mass spectrometry to address clinical concerns. Clin. Mass Spectrom..

[14] Karlsson R., Gonzales-Siles L., Gomila M., Busquets A., Salva-Serra F., Jaen-Luchoro D., Jakobsson H.E., Karlsson A., Boulund F., Kristiansson E. (2018). Proteotyping bacteria: Characterization, differentiation and identification of pneumococcus and other species within the Mitis Group of the genus Streptococcus by tandem mass spectrometry proteomics. PLoS ONE.

[15] Hayoun K., Gaillard J.C., Pible O., Alpha-Bazin B., Armengaud J. (2020). High-throughput proteotyping of bacterial isolates by double barrel chromatography-tandem mass spectrometry based on microplate paramagnetic beads and phylopeptidomics. J. Proteom..

[16] Mesuere B., Willems T., Van der Jeugt F., Devreese B., Vandamme P., Dawyndt P. (2016). Unipept web services for metaproteomics analysis. Bioinformatics.

[17] Padliya N.D., Garrett W.M., Campbell K.B., Tabb D.L., Cooper B. (2007). Tandem mass spectrometry for the detection of plant pathogenic fungi and the effects of database composition on protein inferences. Proteomics.

[18] Verberkmoes N.C., Hervey W.J., Shah M., Land M., Hauser L., Larimer F.W., Van Berkel G.J., Goeringer D.E. (2005). Evaluation of “shotgun” proteomics for identification of biological threat agents in complex environmental matrixes: Experimental simulations. Anal. Chem..

[19] Pible O., Allain F., Jouffret V., Culotta K., Miotello G., Armengaud J. (2020). Estimating relative biomasses of organisms in microbiota using “phylopeptidomics”. Microbiome.

[20] Hayoun K., Gouveia D., Grenga L., Pible O., Armengaud J., Alpha-Bazin B. (2019). Evaluation of Sample Preparation Methods for Fast Proteotyping of Microorganisms by Tandem Mass Spectrometry. Front. Microbiol..

[21] Reasoner D.J., Geldreich E.E. (1985). A new medium for the enumeration and subculture of bacteria from potable water. Appl. Environ. Microbiol..

[22] Mappa C., Pible O., Armengaud J., Alpha-Bazin B. (2018). Assessing the ratio of Bacillus spores and vegetative cells by shotgun proteomics. Environ. Sci. Pollut. Res. Int..

[23] Hartmann E.M., Allain F., Gaillard J.C., Pible O., Armengaud J. (2014). Taking the shortcut for high-throughput shotgun proteomic analysis of bacteria. Methods Mol. Biol..

[24] Perez-Riverol Y., Csordas A., Bai J., Bernal-Llinares M., Hewapathirana S., Kundu D.J., Inuganti A., Griss J., Mayer G., Eisenacher M. (2019). The PRIDE database and related tools and resources in 2019: Improving support for quantification data. Nucleic Acids Res..

[25] Kepler R.M., Luangsa-Ard J.J., Hywel-Jones N.L., Quandt C.A., Sung G.H., Rehner S.A., Aime M.C., Henkel T.W., Sanjuan T., Zare R. (2017). A phylogenetically-based nomenclature for Cordycipitaceae (Hypocreales). IMA Fungus.

[26] Bernard G., Pathmanathan J.S., Lannes R., Lopez P., Bapteste E. (2018). Microbial Dark Matter Investigations: How Microbial Studies Transform Biological Knowledge and Empirically Sketch a Logic of Scientific Discovery. Genome Biol. Evol..

[27] Saw J.H., Spang A., Zaremba-Niedzwiedzka K., Juzokaite L., Dodsworth J.A., Murugapiran S.K., Colman D.R., Takacs-Vesbach C., Hedlund B.P., Guy L. (2015). Exploring microbial dark matter to resolve the deep archaeal ancestry of eukaryotes. Philos. Trans. R. Soc. Lond. B Biol. Sci..

[28] Sheu S.Y., Chen W.T., Young C.C., Chen W.M. (2018). Rheinheimera coerulea sp. nov. isolated from a freshwater creek, and emended description of genus Rheinheimera Brettar et al. 2002. Int. J. Syst. Evol. Microbiol..

[29] Pible O., Armengaud J. (2015). Improving the quality of genome, protein sequence, and taxonomy databases: A prerequisite for microbiome meta-omics 2.0. Proteomics.

[30] Murray A.E., Freudenstein J., Gribaldo S., Hatzenpichler R., Hugenholtz P., Kampfer P., Konstantinidis K.T., Lane C.E., Papke R.T., Parks D.H. (2020). Roadmap for naming uncultivated Archaea and Bacteria. Nat. Microbiol..

[31] Connon S.A., Giovannoni S.J. (2002). High-throughput methods for culturing microorganisms in very-low-nutrient media yield diverse new marine isolates. Appl. Environ. Microbiol..

[32] Chicote E., Moreno D.A., Garcia A.M., Sarro M.I., Lorenzo P.I., Montero F. (2004). Biofouling on the walls of a spent nuclear fuel pool with radioactive ultrapure water. Biofouling.

[33] Sarro M.I., Garcia A.M., Moreno D.A., Montero F. (2007). Development and characterization of biofilms on stainless steel and titanium in spent nuclear fuel pools. J. Ind. Microbiol. Biotechnol..

[34] Rivasseau C., Farhi E., Compagnon E., de Gouvion Saint Cyr D., van Lis R., Falconet D., Kuntz M., Atteia A., Coute A. (2016). Coccomyxa actinabiotis sp. nov. (Trebouxiophyceae, Chlorophyta), a new green microalga living in the spent fuel cooling pool of a nuclear reactor. J. Phycol..

[35] Gallois N., Alpha-Bazin B., Ortet P., Barakat M., Piette L., Long J., Berthomieu C., Armengaud J., Chapon V. (2018). Proteogenomic insights into uranium tolerance of a Chernobyl’s Microbacterium bacterial isolate. J. Proteom..

